# Potential of Copper and Copper Compounds for Anticancer Applications

**DOI:** 10.3390/ph16020234

**Published:** 2023-02-03

**Authors:** Peng Ji, Peng Wang, Hao Chen, Yajing Xu, Jianwen Ge, Zechong Tian, Zhirong Yan

**Affiliations:** 1 Jiangsu Provincial Key Laboratory of Chiral Pharmaceutical Chemicals Biologically Manufacturing, College of Pharmacy and Chemistry & Chemical Engineering, Taizhou University, Taizhou 225300, China; 2 Fujian Key Laboratory of Women and Children’s Critical Diseases Research, Department of Anesthesiology, Fujian Maternity and Child Health Hospital, College of Clinical Medicine for Obstetrics & Gynecology and Pediatrics, Fujian Medical University, Fuzhou 350001, China

**Keywords:** copper, cancer, reactive oxygen species, oxidative stress, apoptosis, copper compounds

## Abstract

Inducing cancer cell death has always been a research hotspot in life sciences. With the continuous deepening and diversification of related research, the potential value of metal elements in inducing cell death has been explored. Taking iron as an example, ferroptosis, mainly characterized by increasing iron load and driving the production of large amounts of lipid peroxides and eventually leading to cell death, has recently attracted great interest in the cancer research community. After iron, copper, a trace element, has received extensive attention in cell death, especially in inducing tumor cell death. Copper and its complexes can induce autophagy or apoptosis in tumor cells through a variety of different mechanisms of action (activation of stress pathways, arrest of cell cycle, inhibition of angiogenesis, cuproptosis, and paraptosis), which are promising in cancer therapy and have become new hotspots in cancer treatment research. This article reviews the main mechanisms and potential applications of novel copper and copper compound-induced cell death, focusing on copper compounds and their anticancer applications.

## 1. Introduction

Surgery, chemotherapy, and radiotherapy, as traditional cancer treatments, bring specific therapeutic effects accompanied by unavoidable side effects [1]. The unique nature of cancer cells (immortal proliferation, transformation, and susceptibility to metastasis) has been a difficult point in cancer treatment. Current conventional therapies are no longer fully applicable, and more advanced treatments must be found. Therefore, methods with fewer side effects and high therapeutic efficacy are being actively sought. Currently, the potential of various metal ions in cancer treatment is being explored, which gives us a bright future for cancer treatment.

**Figure 1:**
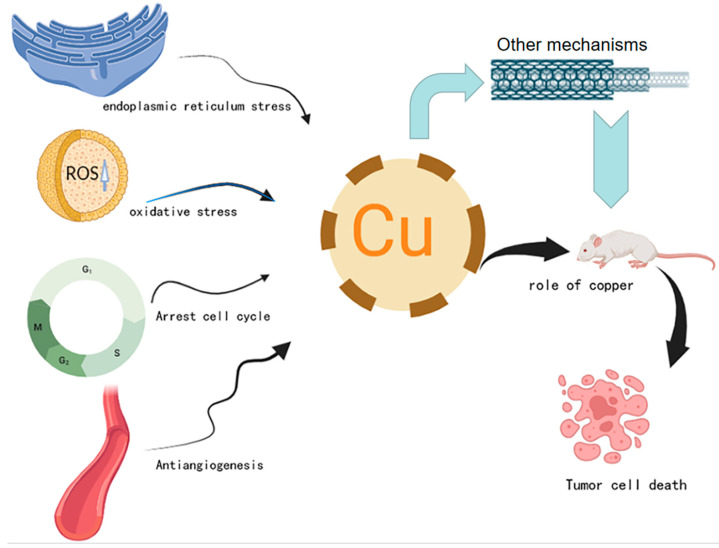
The primary mechanism of copper-induced cell death. Activating stress pathways (endoplasmic reticulum stress and oxidative stress), blocking the cell cycle, and anti-angiogenesis are all used to treat cancer by controlling copper levels and utilizing the cytotoxicity produced by copper excess to kill tumor cells. There are other therapeutic mechanisms to treat cancer by reaching the tumor’s exact location in mice suffering from the tumor and causing tumor cell death under the action of copper.

Copper is an essential element of life, from bacteria and fungi to plants and animals. In humans, copper is an indispensable trace metal element in the body and a necessary cofactor for the organism [2]. It binds to enzymes to assist blood clotting, hormone maturation, and cellular energy processing. The unique redox properties of copper make it both beneficial and detrimental to cells. When the copper content exceeds the everyday needs of the cells, it will produce cytotoxicity and kill the cells; when the copper is deficient, the absorption and transport of copper will be hindered, resulting in an abnormal distribution of copper in each cell [3]. It can be seen that excessive or insufficient copper accumulation will affect the regular physiological activity of cells. Because the growth and metastasis of tumors have a higher demand for copper, a metal nutrient, copper-related diagnosis and treatment methods are very suitable for tumors. According to the unique mechanism of copper, researchers have applied it to tumor cells and found that it can effectively induce tumor cell death. They have designed a variety of copper complexes, which have achieved good results in tumor treatment [4]. The current research progress on the induction of autophagy and apoptosis in tumor cells by copper and its complexes and their mechanisms (activation of stress pathways, arrest of cell cycle, and inhibition of angiogenesis) are summarized as follows (Figure 1).

## 2. Distribution and Importance of Copper

It is well known that the copper ion is an essential micronutrient for the human body. A copper intake of 0.8 mg/d is sufficient to maintain and regulate the copper balance in the human body [5]. Most of the copper ingested by the human body from food is Cu^2+^, which cells cannot absorb and utilize. Generally, it needs to be processed by digestive tract cells. There is a variety of reductases on the surface of these cells. With the assistance of divalent metal transporter 1 (DMT1), Cu^2+^ can be reduced to Cu^+^ and combined with copper transporter 1 (CTR1) to enter the cells. The Cu^+^ that enters the cell is delivered to the copper chaperone protein antioxidant 1 (Atox1), which combines with the copper transport ATPase β (ATP7β) through the Golgi complex pathway and preceruloplasmin to produce ceruloplasmin (CP) [6], which further acts on organs and tissues throughout the body.

Copper has important redox activity and can participate in various biochemical reactions by accepting and donating electrons. As a trace element, copper is widely distributed in biological tissues, participates in the formation of proteins and related physiological regulation, and plays a vital role in the human body [6]. The function of some proteins requires the activation of copper, such as cytochrome c oxidase (COX), NADH deoxygenase-2 (ND2), and other essential proteins in the body; the role of copper is irreplaceable [7]. Copper, combined with various proteins or enzymes, can also participate in crucial life processes in the body, such as energy metabolism, oxidative detoxification, and mitochondrial respiration. The absorption, transport, storage, and excretion of copper ions in the body are the basic processes for stabilizing copper metabolism. Intracellular copper is strictly regulated, on the one hand, to supply sufficient copper proteins and, on the other hand, to prevent toxic oxidative stress. Studies have shown that an excessive accumulation of intracellular copper can trigger the accumulation of mitochondrial lipid-acylated proteins and interfere with the role of Fe-S cluster proteins [8]. The reasonable control of the toxicity of copper can maximize its function.

In addition, there is a strong link between copper and cancer cells. Studies have shown that copper is cytotoxic to cancer cells, has anti-angiogenic, cell cycle growth-inhibiting, and energy metabolism-regulating effects in anticancer therapy, and its copper complexes can also produce antibacterial, antiviral, anti-inflammatory, antitumor, and enzyme-inhibiting activities, which can play an essential role in cancer therapy [9,10,11,12]. Based on this feature, copper has been continuously introduced into anticancer drugs. It is of great importance for cancer treatment.

## 3. Mechanism of Copper Compound-Induced Cell Death

Given the high redox activity of copper, it is tightly controlled in the complex physiological environment of the body, so it is usually not present as a free ion but is bound to the corresponding protein. Representative copper-dependent proteins are essential for copper transport and cancer therapeutic processes. In the future, developing drugs based on copper-dependent proteins may be a helpful direction for cancer treatment. The liver is a significant organ for copper deposition, and most accumulated hepatic copper is bound to metallothionein (MT), a metal-rich, cysteine-rich, low-molecular-weight protein [13]. MT has a high affinity for metals and protects against metal toxicity [14]. Mammalian ATP7A and ATP7B isoforms play a crucial role in cellular physiology. For example, dietary copper involved in transfer enters the bloodstream via intestinal epithelial cells, participates in reabsorption with hepatic bile, and is finally excreted, maintaining intracellular copper levels below toxic levels. In addition, they retain homeostasis in tissues such as systemic copper absorption, copper excretion, and copper in the brain. Cu, Zn-superoxide dismutase (SOD) is a water-soluble copper–zinc-structured enzyme protein present in the cytoplasm of eukaryotic cells and is a homodimeric enzyme that catalyzes the disproportionation of two identical molecules in different directions; the enzyme has a vital defense role against reactive oxygen species generated by cellular metabolism, which are responsible for the production of membrane lipid peroxidation and damage to DNA and proteins (oxidative stress) [15]. CCS is a copper chaperone protein of SOD in the cytoplasm with additional functions in the cell. CCS enters the nucleus and regulates the HIF-1 transcriptional complex in a copper-dependent manner. This leads to vascular endothelial growth factor (VEGF) expression, promoting tumor growth [16].

### 3.1. Activation of Stress Pathways

Integrated stress response (ISR) is a self-rescue response of cells under various stress stimuli, which is a crucial stress support pathway to promote the adaptation of cells to the stress from the tumor microenvironment [17]. ISR is an evolutionarily conserved mechanism that allows communication between organelles, including mitochondria and the nucleus. ISR slows protein synthesis in the cytoplasm and alters the transcriptome in the nucleus following mitochondrial stress. With the help of the ATF4 transcription factor, it promotes metabolic rewiring, amino acid, and antioxidant synthesis to counteract cellular stress. Under chronic stress, ISR leads to apoptosis. This pathway intersects at the eukaryotic initiation factor subunit eIF2α to slow translation and protein synthesis while allowing adaptive mechanisms to continue [18,19]. Various physiological and pathological changes, including endoplasmic reticulum stress, amino acid deficiency, viral infection, oxidative stress, etc., can activate ISR. The activation of ISR can disrupt the homeostasis of cancer cells, and oxidative stress and endoplasmic reticulum stress have broad application prospects in activating stress pathways to treat cancer [20].

ROS, or reactive oxygen species, refers to the harmful compounds produced during the oxidation reaction of the body. ROS plays a critical role in the biohomeostasis and pathogenesis of human diseases, including cancer. Cancer cells maintain a delicate balance between ROS and antioxidants, contributing to pathogenesis and clinical challenges by targeting several signaling pathways that converge with cancer hallmarks [21]. Under the influence of various factors, such as pressure, ultraviolet, radiation, ischemia/reperfusion, and inflammation, the body produces too many ROS, leading to oxidative stress [22]. FDX1 is vital in the principle of the copper-induced activation of cell death reduction. It acts as a copper ion carrier-induced cell death ferric oxygen-reducing protein factor that reduces Cu^2+^ to Cu^+^, which is more copper toxic and induces cell death. In addition, Tsvetkov P et al. found that FDX1 is an upstream regulator of protein lipoic modification in their study of copper-targeted TCA cyclin-induced cell death [4]. The reduction signaling of the copper-induced cell death mechanism involves the formation of cysteine (Cys) residue(s) adducts [23]. Under reduction-activated signaling, ROS binds the relevant regulatory signals to redox mechanisms to induce autophagy and apoptosis in tumor cells. Oxidative stress is an imbalance between the body’s production and the scavenging of oxygen-free radicals. When the body changes the levels of oxidants and antioxidants, and many oxidative damage markers appear, it may indicate certain diseases, such as cancer [24]. Generally, ROS are the leading cause of oxidative stress. Under physiological conditions, the concentration of ROS is deficient, and it is a vital substance involved in signal transduction. It can regulate various transcription factors and proteins in the body and then affect a wide range of signal pathways related to metabolic reaction, inflammatory reaction, DNA repair, growth promotion or differentiation, apoptosis, autophagy, etc. When the number of ROS in the body is too high, oxidative stress can damage lipids, proteins, and DNA, whereas peroxides can induce apoptosis [25]. Excessive copper in the cell will generate a large number of ROS in a short or long time to produce cytotoxicity, thus triggering various apoptoses. The Fenton reaction is an inorganic chemical reaction in which divalent iron ions (Fe^2+^) catalyze hydrogen peroxide (H_2_O_2_) to produce toxic hydroxyl radicals (·OH), which trigger cellular lipid peroxidation. The Haber–Weiss reaction is one of the types of Fenton reactions [26]. In addition to iron, copper can also induce the same response. The presence of copper will also lead to the conversion of hydrogen peroxide to hydroxyl radicals, considered the most reactive and destructive ROS [23]. In addition, copper also participates in the Haber–Weiss reaction, which catalyzes the formation of ROS, and then ROS will lead to membrane lipid peroxidation [27]. Based on this feature, when the concentration of copper in the cells is increased, it will induce the death of cancer cells and become one of the strategies for developing anticancer drugs.

Guo et al. designed a new copper (II) complex and evaluated it cytologically. The results showed that the intracellular ROS level significantly increased after the cells were treated with the new copper (II) complex. It was found that the compound could effectively kill HeLa cells through the autophagy pathway triggered by ROS [28]. Liu et al. synthesized nano-copper/catechol-based metal-organic frameworks (CuHPT) by coordinating copper ions with catechol groups, which can oxidize glutathione (GSH) into glutathione disulfide, and at the same time, coordinate copper is reduced and decomposed. Subsequently, catechol ligands and reduced copper ions released from the formulations can synergistically enhance the production of intracellular ROS through the Fenton-like reactions of autooxidation and the depletion of GSH. The results show that CuHPT can effectively inhibit the growth of drug-resistant tumor cells and use the high content of GSH in drug-resistant cancer cells as a “trigger” to activate and maintain the amplification effect of ROS, which can effectively kill drug-resistant cancer cells, showing excellent anti-tumor results [29].

**Figure 2:**
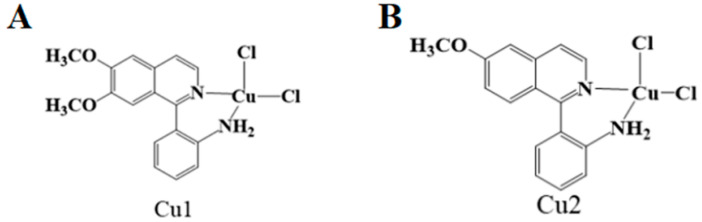
Chemical structure of Cu1 (**A**) complex and Cu2 (**B**).

In addition to causing oxidative stress, excessive copper causes endoplasmic reticulum (ER) stress, damaging DNA and inhibiting cell proliferation [30]. In tumor cells, multiple intrinsic and extrinsic cellular stresses disrupt intracellular proteostasis. As the central organelle for protein quality control, the endoplasmic reticulum controls protein homeostasis through the precise adaptive regulation of related mechanisms. However, when protein homeostasis is disrupted, protein misfolding results in insufficient endoplasmic reticulum corrective capacity, and regulatory signals may shift from adaptive to pro-apoptotic and the clearance of damaged cells. Therefore, different regulatory modes under ER stress determine the fate of tumor cells [31]. Under the action of copper, the permeability of the endoplasmic reticulum is improved, and protein misfolding will inhibit the corrective ability of the endoplasmic reticulum; the number of ROS increases, and the regulatory signal becomes pro-apoptotic and removes damaged cells, resulting in cell apoptosis [32]. Gul et al. combined the ligands “L1” 2-(6,7-dimethoxyisoquinolin-1-yl) aniline and “L2” 2(6-methoxyisoquinolin-1-yl) with aniline CuCl_2_·2H_2_O in the solvent of dichloromethane and methanol (v:v = 1:1) to synthesize two copper complexes, Cu1 (Figure 2A) and Cu2 (Figure 2B) [33]. The study found that these two complexes showed high toxicity to tumor cells after treating A549 cancer cells and normal human cells. Still, the toxicity to normal human cells is minimal, indicating that it has a good effect on treating tumors. The experiments also found that ROS exhibited less co-localization and more diffusion with increasing concentrations of Cu complexes. The endoplasmic reticulum membrane was more permeable, leading to the induction of ER-mediated apoptosis. Western blot analysis further confirmed this pathway.

### 3.2. Arresting the Cell Cycle

**Figure 3:**
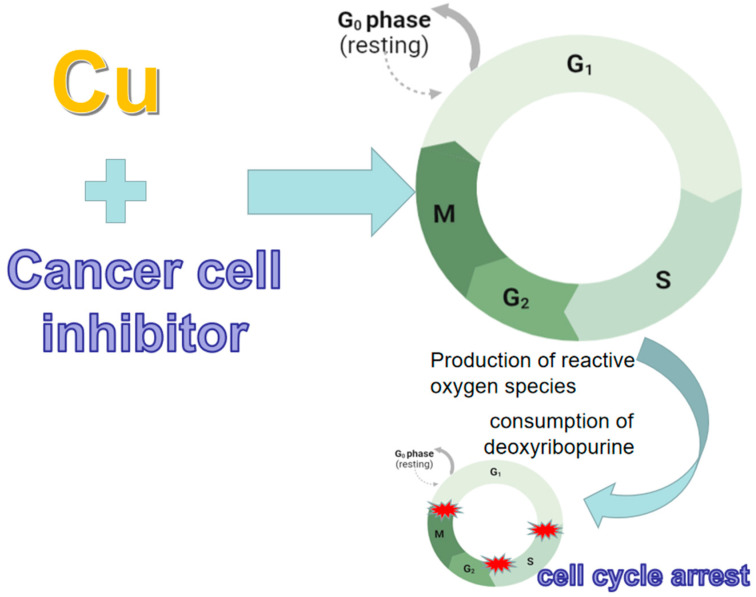
Copper-binding inhibitors induce reactive oxygen species production and deoxy ribosyl purine depletion. Treatment of cancer cells with copper in combination with NSC319726 enhances the inhibitory effect of this compound on cancer cells, leading to the intracellular production of reactive oxygen species and the depletion of deoxyribonucleopurines (e.g., 20-deoxyguanosine), inducing cellular G-phase arrest and apoptosis.

Cell cycle regulation requires the participation of many signals, and the loss of signals can lead to cell cycle arrest, thereby inhibiting cell proliferation and inducing apoptosis [34]. The cell cycle is a series of tightly controlled processes. Eukaryotic cells undergo cell division through mitosis. When the cell cycle is out of control, it may lead to the proliferation of tumor cells. There are many types of tumors, and they seriously threaten human life. The cell cycle is closely related to the pathogenesis of tumors. Therefore, starting from the mechanism of cell cycle regulation has a significant theoretical value in biology and has important guiding significance for preventing and treating some cell cycle-related diseases. The three stages, G1-S, S, and G2-M, are the three main checkpoints of the cell cycle [14]. Cell cycle arrest is a crucial mechanism for many anticancer drugs to inhibit tumor cell proliferation [35]. NSC319726 is an effective cancer cell cycle growth inhibitor; Shimada et al. found that copper dysregulation can induce DNA damage, resulting in cell cycle arrest [36]. The combination of NSC319726 with metal ions can cause cancer cell death, especially over metals. After binding with various metal ions, studies found that only copper ions significantly enhanced the inhibitory effect of this compound on cancer cells. Copper binding to NSC319726 treats cancer cells, resulting in the production of intracellular reactive oxygen species and the depletion of deoxyribopurines (such as 20-deoxyguanosine), inducing cell G-phase arrest and apoptosis (Figure 3). These results suggest that DNA damage is a significant consequence of copper-mediated oxidative stress, which subsequently causes cell cycle arrest. Therefore, copper dysregulation and metal-induced DNA damage may result from exposure to specific therapeutic agents or exogenous substances. 

Multiple myeloma (MM) is a malignant plasma cell disease that belongs to one of the B-cell lymphomas. As an incurable hematological malignancy, it originates from plasma cells in the bone marrow. The current treatment for MM mainly includes conventional chemotherapy, immunotherapy, hematopoietic stem cell transplantation, etc. Bortezomib and lenalidomide are also representative drugs for the treatment of MM. MM cells are highly heterogeneous, and the complex tumor microenvironment that can be formed between MM cells and bone marrow cells makes drug resistance in MM treatment a bottleneck that is difficult to break through, and the recurrence of MM will increase the tumor load, enhance drug resistance, and shorten the remission period. Therefore, the treatment of MM needs to open up more therapeutic avenues. Disulfiram (DSF) has strong copper dependence and toxicity to cancer cells. Xu et al. found that the DSF/Cu complex induced apoptosis in MM cells and MM progenitor cells and significantly induced cell cycle arrest in the G2/M phase in MM.1S and RPMI8226 cells. Furthermore, JC-1 and protein blotting showed that DSF/Cu disrupted mitochondrial membrane integrity and cleaved cystatin-8 in MM cells, indicating that it activated exogenous and intrinsic apoptotic pathways. DSF/Cu significantly reduced the tumor volume and prolonged the overall survival of MM mice compared with the control group. DSF/Cu exhibited potent anti-myeloma activity in vitro and in vivo, highlighting the valuable clinical potential of DSF/Cu in MM treatment [37].

### 3.3. Anti-Angiogenesis

Angiogenesis is the initial process of tumor cell proliferation and metastasis. Blood vessels provide numerous nutrients for tumor cell growth, whereby tumor cells acquire the ability to enter a stage of rapid growth and exhibit increased metastatic potential, which is a significant cause of morbidity and progression in cancer patients [38]. Copper is a trace element necessary for the secretion of several angiogenic factors and acts as an essential cofactor in angiogenesis. Many copper-dependent enzymes also play a significant role in cell proliferation and migration [39]. Tumors may promote angiogenesis by upregulating copper-carrying proteins and pathways. 

Tumor immunotherapy is a method to stimulate the immune system to restart so that the body can recover and maintain the anti-tumor immune response. It has become a breakthrough in treating various cancers and brings opportunities for cancer treatment. Unfortunately, these immunotherapies are only suitable for a subset of patients, and some patients only show transient therapeutic effects [40]. Copper depletion can turn off the “angiogenic switch”, preventing the expression of vascular endothelial growth factors, or its impact can inhibit the formation of tumor blood vessels, cut off the supply of oxygen and nutrients to tumor cells, and thus starve the tumor. Pan et al. found that thiomolybdate (TM) can safely induce a copper deficiency in humans and mice. This copper deficiency can significantly damage tumor cells and angiogenesis with minimal side effects [41]. Experiments show that TM can reduce in vitro: (1) vascular endothelial growth factor; (2) fibroblast growth factor 2/basic fibroblast growth factor; (3) interleukin (IL)-1α; (4) IL-6; and (5) the production of five pro-angiogenic mediators, IL-8. The Cu-mediated antiangiogenic effect is highly effective and, when combined with other cancer therapies or new vascular targeting mechanisms, will result in enhanced antitumor efficacy.

### 3.4. Cuproptosis

Copper plays a pivotal role as a cofactor necessary for the life activities of living organisms. The average concentration of copper ions in the body must be kept low and in dynamic equilibrium. Once the concentration exceeds the threshold set by the body’s homeostasis, cytotoxicity can occur. Interestingly, this toxicity from copper overload can induce cell death even when attempts are made to block the currently known modes of cell death. This phenomenon is somewhat similar to the previously studied case of ferroptosis due to iron accumulation, and the researchers officially named this mechanism of copper ion-induced cell death “cuproptosis” [4]. Cuproptosis is distinguished from previously programmed deaths, including ferroptosis, by a strong link between the regulatory process and mitochondria. Excess copper ions induce cell death through direct binding to thioredoxin, a lipidated component of the mitochondrial tricarboxylic acid (TCA) cycle pathway, leading to the abnormal aggregation of thioredoxin and the loss of iron–sulfur cluster proteins in the respiratory chain complex, which ultimately leads to a proteotoxic stress response. Studies on the mechanisms of cuproptosis-induced cell death have focused on studies on copper ion carriers, which bind copper ions and are responsible for their transport between cells [42]. The treatment of copper ion carriers using known inhibitors of cell death (including ferroptosis, oxidative stress, etc.) did not affect their induction of cell death, suggesting that the mechanism of copper ion carrier-induced cell death is distinct from known mechanisms of cell death regulation. Fortunately, the researchers found that cells that depend primarily on mitochondrial respiration are susceptible to copper ion carriers. The treatment of cells with inhibitors of mitochondrial respiratory pathways related to mitochondrial fatty acids, inhibitors of mitochondrial function, and antioxidants had significant effects on copper ion carriers, and copper ions may not directly affect the electron transport chain but rather the TCA. Further studies of the TCA process identified FDX1 and protein lipoic acidification as critical regulators of copper ion-induced cuproptosis. FDX1 and protein lipoic acidification-related enzymes (LIPT1, LIAS, DLD) can inhibit cuproptosis in cells, and FDX1 is an upstream regulator of protein lipoic acidification [4]. With the high expression of lipoic acidulated mitochondrial proteins in cancer and the increased respiration of cancer cells, using copper ion metal carriers to kill cancer cells will potentially be a new approach to treating cancer.

### 3.5. Paraptosis

Paraptosis is a non-apoptotic programmed cell death, also known as type III programmed cell death (PCD). In 2000, Sperandio et al. found a form of death different from conventional apoptosis in 293T cell lines overexpressing insulin-like growth factor 1 receptor (IGFIR), which can be considered necrosis in some cases and defined as paraptosis [43]. At present, studies on paraptosis still mainly focus on cell morphology, which shows increased cytoplasmic density, the vacuolization of the cell pulp, the swelling of mitochondria and endoplasmic reticulum, but no nuclear sequestration phenomenon, irregular aggregation in late stages, seam-like cells forming multi-membrane vesicles, and eventual phagocytosis by macrophages without causing inflammatory responses in surrounding tissues [44]. The four commonly used assays for paraptosis cells are (1) DNA in situ nick transfer (ISEL) and DNA in situ end labeling (TUNEL); (2) agarose gel electrophoresis to observe the “stepped DNA bands” produced by the degradation of chromatin DNA by ribosomal endonucleases; (3) apoptosis inhibitor assay; (4) cell regulation enzymatic analysis. In recent years, it has been found that some copper complexes can induce paraptosis in vitro by the mechanism that copper complexes can inhibit proteasome activity in tumor cells and play an inhibitory reproductive role in the accumulation of misfolded proteins while being able to induce endoplasmic reticulum homeostasis to play the same function [45]. In addition, it was also found that with the 4-amino-1,4-dihydro-3-(2-pyridyl)-5-thioxo-1,2,4-triazole ligand called A0, A0 had an inhibitory effect on human fibrosarcoma HT1080, and A0-treated tumor cells exhibited significant vacuolization and alteration of endoplasmic reticulum morphology, causing paraptosis in tumor cells, which was comparable to the chemotherapeutic drug cisplatin equal effect [44]. Therefore, the induction of paraptosis in cancer cells is also an effective strategy for developing copper drugs.

### 3.6. Others

With the rapid development of nanomedicine materials, many novel, low-toxicity, and high-efficiency cancer treatments have been developed to treat advanced or more difficult-to-treat drug-resistant cancers, such as photothermal therapy (PTT), photodynamic therapy (PDT), or chemo-dynamic therapy (CDT), and other new therapies have shown strong tumor treatment prospects [46]. PTT uses materials with high photothermal conversion efficiency, injects them into the human body, makes them gather in tumor tissue, and converts light energy into heat energy under the irradiation of an external light source (generally near-infrared light) to kill cancer cells. It has the characteristics of high selectivity, minor systemic side effects, short treatment time (about a few minutes), and apparent therapeutic effects [47]. However, intense laser light can increase the local temperature. If the tissue temperature exceeds 60 °C, protein denaturation and cell membrane disruption will instantly cause cell death. PTT kills tumors, injures innocents, and burns normal human tissue near cancer. The treatment effect will be affected by lowering the temperature [48]. The combination of nanomedical materials and their treatment can focus the high temperature on tumor cells, which is an excellent solution to this problem. Cu nanomaterials show strong localized surface plasmon resonance absorption in the NIR spectral range. They can convert the absorbed light energy into thermal energy well, so copper-based nanomaterials have a good prospect in applying photothermal therapy [49]. Another effective method is CDT, which is a promising therapeutic mode. It utilizes the endogenous overexpression of hydrogen peroxide (H_2_O_2_) in tumors to generate toxic hydroxyl radicals through Fenton/Fenton-like reactions catalyzed by metals (Cu^+^, Fe^2+^, Cu^+^, Mn^2+^, Mo^4+^, W^4+^, Ti^3+^, etc.). Compared with other ROS therapies, CDT has the advantages of higher catalytic performance to generate ROS, less dependence on external stimuli, deep tissue therapeutic ability, and resistance to drug resistance [50]. Studies have shown that Cu+ ion-mediated Fenton-like reactions have kinetic and thermodynamic advantages in mildly acidic TMEs. Cu-organic complexes are attractive and potential alternatives to traditional Fe-based complexes [51].

In summary, all these mechanisms can be excellent in targeting to kill tumor cells with less damage to normal human cells, and based on these therapeutic mechanisms, we found some well-acting copper complexes that can accomplish mechanisms such as cell cycle blocking and anti-angiogenesis to cause tumor cell death [52], which have good prospects for application.

## 4. Application of Novel Copper Complexes in Tumor Therapy

### 4.1. Application of Copper-Activated Stress Pathway in Cancer

#### Disulfiram/Cu Complex

**Figure 4:**
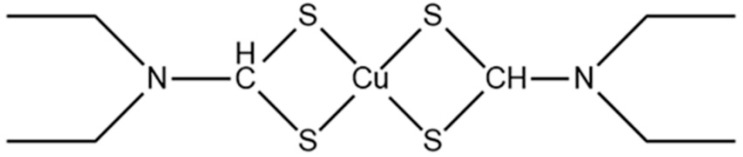
Chemical structure of disulfiram/copper complexes.

Acute myeloid leukemia (AML) is a highly lethal hematological malignancy. Chemotherapy is a widely used therapeutic method in clinics. However, chemotherapy is faced with thorny problems such as poor selectivity, toxic side effects on normal tissues, and easy recurrence and metastasis of patients [53]. Therefore, it is of great significance to explore new therapeutic strategies. The study has found that disulfiram has specific anti-tumor activity. The disulfiram/copper complex (Figure 4) can effectively reverse the drug resistance of doxorubicin (ADM)-resistant acute leukemia cell lines by inducing apoptosis. The IC50 value is significantly lower than that of disulfiram, indicating that copper can dramatically improve the effect of disulfiram on inducing tumor cell apoptosis, which is an excellent signal for treating acute leukemia [54,55]. Research on the mechanism of the disulfiram/copper activating stress pathway shows that the disulfiram/copper complex may induce apoptosis in sensitive leukemia cell lines by activating the MAPK pathway [55]. Activating the MAPK pathway can effectively improve the sensitivity of ADM to drug-resistant HL60/ADM and reverse drug resistance. In addition, the MAPK signaling pathway can be activated in various cancers, including gastric cancer [56]. Targeting the MAPK pathway is considered a promising cancer treatment strategy. Currently, the relevant research on the copper treatment of AML is still at the molecular level. Further in-depth mechanism research is needed to provide a practical solution for the future clinical application of AML treatment.

Gastric cancer is one of the common malignant tumors of the digestive tract. Due to the lack of apparent symptoms in the early stage, gastric cancer patients are generally detected late. About 80% of gastric cancer patients are diagnosed in the middle and advanced stages, which is very difficult to treat [57]. Most clinical treatment is based on surgery, supplemented by radiotherapy and chemotherapy. The cure rate is meager, the limitations are relatively large, and chemotherapy drugs are prone to drug resistance and side effects. Because gastric cancer has heterogeneous characteristics, even the current research on new gene therapy and immunotherapy cannot effectively treat gastric cancer and improve the survival rate of patients [58]. Therefore, developing new therapeutic strategies to improve the current status of gastric cancer treatment is of great significance. Studies have found that disulfiram metabolites have been shown to complex with copper ions and can effectively inhibit copper-dependent enzymes (such as superoxide dismutase to protect against oxidative stress), matrix metalloproteinases (promoting cancer cell invasion and metastasis) or dopamine β-Monooxygenase [59], etc., and then achieve the killing of cancer cells. In addition, disulfiram/copper complexes can also induce DNA damage and regulate energy metabolism, thereby achieving the effect of treating tumors [60,61,62]. The disulfiram/copper complex has been shown to inhibit gastric cancer cell proliferation effectively. The mechanism may be a pro-oxidative environment, which generates ROS and induces the programmed cell death of gastric cancer cells, thereby achieving the purpose of treating gastric cancer. Therefore, the disulfiram/copper complex has good potential for treating gastric cancer.

### 4.2. The Application of Cell Cycle Arrest in Cancer

#### 4.2.1. New Copper Complexes Containing Naphthyl Groups

**Figure 5:**
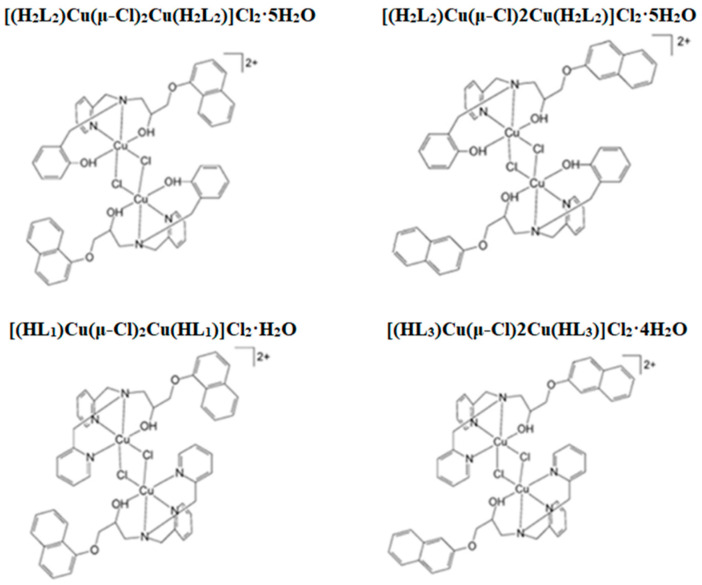
Chemical structure of [(H_2_L_2_)Cu(μ-Cl)_2_Cu(H_2_L_2_)]Cl_2_·5H_2_O, [(H_2_L_2_)Cu(μ-Cl)2Cu(H_2_L_2_)]Cl_2_·5H_2_O, [(HL_1_)Cu(μ-Cl)_2_Cu(HL_1_)]Cl_2_·H_2_O, [(HL_3_)Cu(μ-Cl)2Cu(HL_3_)]Cl_2_·4H_2_O.

The antitumor activity of the new di-iron(III) complexes containing the naphthyl group found that the new iron complexes containing the naphthyl group could induce apoptosis in cancer cells [63]. The study found that HL_1_ and HL_2_ with a naphthyl side chain are isomers of each other, and the complex of HL_1_ has higher activity than the complex containing HL_2_ and has a more substantial inhibitory effect on leukemia cells. Based on the research idea of the antitumor activity of the new iron complexes, Karthick et al. studied four new ligands containing naphthyl and the corresponding new copper complexes. They found that the new copper complexes containing naphthyl can also promote leukemia cell apoptosis [64]. This experiment combined four new ligands (HL_1_, H_2_L_2_, HL_3_, H_2_L_4_, Figure 5 with naphthyl side chain units with copper ions to form new copper complexes. The interaction mechanism between copper complexes and leukemia cells was observed by cell cycle analysis, cysteine protease activation, and other methods. It was found that the HL_1_ and HL_3_ ligands containing the naphthyl group and their copper complexes had positive effects on leukemia cell lines THP-1 and U937. The target compound was first activated as caspase-8, promoting the activation of subsequent caspase-3 (effector), leads to the simultaneous enhancement of mitochondrial damage and apoptotic signaling. With higher sensitivity, the number of leukemia cells in the G1 phase was significantly reduced under the action of this type of copper complex. Flow cytometry was used to analyze the effect of copper complexes formed by H_2_L_2_ and H_2_L_4_ on human leukemia cells; this type of copper complex can also hinder the average cycle growth of leukemia cells. It can be seen that the new copper complexes containing the naphthyl group can inhibit the growth and proliferation of leukemia cells through the mechanism of arresting the tumor cell cycle.

#### 4.2.2. Heteromeric Copper(II) Complexes

**Figure 6:**
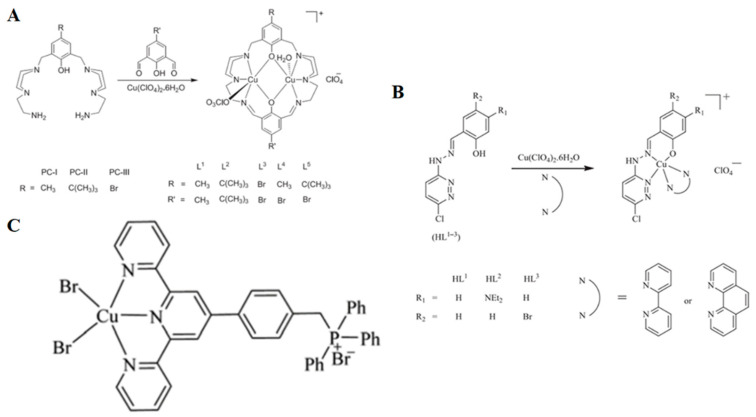
Chemical structure of [Cu_2_L^1–5^(ClO_4_)](ClO_4_) (**A**), chemical structure of [Cu(L^1^)(phen)](ClO_4_) and [Cu(L^2^)(bpy)](ClO_4_) (**B**) and chemical structure of copper(II) tripyridine complex (**C**).

Inspired by traditional metal–organic coordination chemistry, the researchers propose a new concept of constructing Cu nanostructures using metal coordination-driven self-assembly. Karthick et al. synthesized a series of new [Cu_2_L1-5 (ClO_4_)] (ClO_4_-1-5) (Figure 6A)-type macrocyclic binuclear copper (II) complexes through the template condensation reaction between the precursor compound 2,6-bis (4-aminoethylpiperazin-1-ylmethyl)-4-substituted phenol and 2,6-diformyl-4-substituted phenol [65]. The in vitro cytotoxicity of the complex against human epidermoid cancer cells (A431) was tested by MTT assay, and the results showed that the complex has effective anticancer activity. The living cells and fluorescence imaging of A431 cells revealed that the complex induces cell death by blocking DNA replication and apoptosis. Pyridazine is a heterocyclic diazine with a wide range of pharmacological activities, such as antidepressant, antibacterial, and antitumor [66]. Rafi et al. used pyridazine as a ligand to synthesize hydrazine pyridazine ligands through a condensation reaction. Then, they formed a heterocoordinate copper(II) complex [3-chloro-6-(4-diethylaminosalicylhydrazinyl) pyridazine] with copper(II) perchlorate hexahydrate. The inhibitory properties of copper(II) complexes on breast cancer cell proliferation were detected by MTT assay. The experiments showed that the complexes exhibited a significant proliferation inhibition effect on the human breast cancer cell line (MCF-7), and the cell viability was affected by the complexes [67]. The number of breast cancer cells decreased with increasing copper complex concentration. Flow cytometry with propidium iodide staining was used to determine the cell cycle distribution of breast cancer cells in copper(II) complexes. It was found that the proportion of MCF-7 cells treated with copper(II) complexes was significantly increased in the S phase and the G0 phase. The apoptosis induced by the complex in the MCF-7 cell line was analyzed by Hoechst staining, which indicated that the complex had high apoptotic activity. Therefore, copper(II) complexes have anti-tumor effects by causing DNA damage and inducing apoptosis (Figure 6B) [68]. Gu et al. prepared four terpyridine copper(II) complexes (Figure 6C), which exhibited higher cytotoxic activity against several tested cancer cell lines, especially BEL-7402 cells. They showed low toxicity to normal human liver cells [69]. Mechanistic studies have shown that copper complexes can induce G0/G1 phase arrest and alter the expression of cell cycle-related proteins. They also up-regulated Bax and down-regulated Bcl-2 expression, led to the release of cytochrome c and caspase cascade activation, and induced mitochondrial-mediated apoptosis, showing good anti-tumor activity in the mouse xenotransplantation model of BEL-7402 tumor cells.

### 4.3. Application of Anti-Angiogenesis in Cancer

#### 4.3.1. RPTDH Nanoparticles

Targeted nanoparticles target tumor cells, and applying nanoparticles in anti-tumor drug delivery has attracted much attention. The synthesis of metal nanoparticles has attracted much attention due to their unique physical properties, such as high surface area-to-volume ratio, ease of synthesis, facile surface chemistry, and extensive optical properties [70]. The synthesis of metal nanoparticles for improved therapeutic index and drug delivery is becoming an attractive strategy for mainstreaming cancer therapeutic research. During the formation of new blood vessels, the strong demand for copper content is a hallmark of cancer development [71]. Therefore, the development of targeted agents to accurately deliver to cancer cells and significantly reduce the intracellular copper content can inhibit the growth of tumor angiogenesis and achieve starvation therapy for tumors. Copper plays a vital role in tumor angiogenesis, and copper chelation effectively inhibits tumor growth and metastasis by inhibiting tumor angiogenesis [72]. Zhou et al. successfully synthesized a copper-chelating coil-assembled block copolymer RGD-PEG-b-PGA-g-(TETA-DTC-PHis) (RPTDH) through a multi-step chemical reaction [73]. RPTDH has a strong copper chelating ability. Its molecular structure contains specific copper-chelating functional groups triethylenetetetramine bis(sodium dithiocarbamate) and pH-responsive polyhistidine side chains, which can be self-assembled to form spherical nanoparticles with pH-responsive disintegration properties. Intermolecular hydrophobic interactions were used to prepare nanoparticles RPTDH/R848 loaded with TLR7 and TLR8 agonist resiquimod (R848) and exhibited a greatly accelerated release in weakly acidic media mimicking the tumor microenvironment. RPTDH/R848 nanoparticles effectively inhibited HUVECs by blocking the supply of copper ions through copper chelation mobility, invasion, and vascular tube formation, indicating their anti-angiogenic solid activity in vitro. In addition, RPTDH/R848 nanoparticles significantly induced the maturation and activation of human plasma cell CAL-1 cells, indicating their immune activating ability. In mammary tumor-bearing mice, RPTDH/R848 nanoparticles showed a good targeting ability to both primary mammary tumors and lung metastases and further substantially inhibited tumor growth and metastasis through copper deficiency-induced anti-angiogenesis and R848-induced immune activation. In conclusion, RPTDH/R848 nanoparticles can be used as a therapeutic agent for metastatic breast cancer through anti-angiogenesis and immune activation.

#### 4.3.2. VEGF73-101/Cu(II) Complex

In tumor occurrence and development, many new blood vessels will be formed. These new blood vessels provide the nutrients and water needed for the growth of tumors and, at the same time, spread tumor cells to distant places, forming new metastases in different parts of the body [74]. Copper plays an essential regulatory role in many pathologies involved in angiogenesis [75]. Copper homeostasis is altered in carcinogenesis, tumor progression, and angiogenic diseases. Although many details of the pathways involved remain unclear, some copper-specific ligands have been successfully used as therapeutic agents [76]. Copper-binding peptides that can regulate angiogenesis are a possible way to evaluate the value of new drugs. Tardito et al. found that copper complexes have a unique proteasome inhibition ability and play an indispensable role in cell neovascularization, which opens a new idea for treating tumors. Vascular endothelial growth factor (VEGF) can support copper complexes to participate in angiogenesis and enhance the application of copper complexes in anti-angiogenesis therapy [44]. VEGF73-101 is a fragment of vascular endothelial growth factor (VEGF165), and Zimbone et al. synthesized the VEGF73-101/Cu(II) complex. The anti-angiogenic effect of the complex was evaluated on a human umbilical vein endothelial cell model by cell viability and cell function assays [77]. The results show that copper ions can promote apoptosis and decrease the cell viability of human umbilical vein endothelial cells. The VEGF73-101/Cu(II) complex has an excellent anti-angiogenic effect on the cell membrane model and can be used as an anti-angiogenesis drug in tumor cell therapy.

#### 4.3.3. Topoisomerase Inhibitors

Topoisomerase (topo) is a crucial enzyme indispensable for cellular DNA replication or transcription and is widely found in living organisms. As the reverse rotation of DNA during replication can produce tangles, positive and negative superhelices, etc., which affect the replication of DNA, in order to ensure normal replication, it must rely on the participation of topo for DNA cleavage, gyration, and rejoining, so that the DNA can be successfully deconvoluted, replicated, and transcribed [78]. Compared to normal cells, topoisomerases exhibit high expression levels in tumor cells independent of other factors. Drugs that inhibit topo activity can be divided into DNA embedding agents and non-embedding agents. These topo inhibitors inhibit DNA axis rotation or reduce the enzymatic activity of topo, thereby stabilizing the topo–DNA covalent complex. Topo-I inhibitors inhibit enzymatic catalysis and prevent superhelical DNA from unspinning, which induces DNA strand breaks, promotes cell cycle arrest, inhibits cell division, and ultimately triggers apoptosis. Jinxu Qi et al. showed that metal complexes of aminothioureas have a significant inhibitory effect on the activity of topo-II α [79]. In the absence of metal ligands, the movement of topo-II α is weaker. Still, the complexes formed by pyridoxal condensed aminothioureas with metal ions such as copper can effectively inhibit topo. Still, inevitably, this is also very toxic to normal cells; in any case, these findings will make a significant contribution to the development of drugs with less toxicity and better antitumor effects.

### 4.4. Cuproptosis in Cancer

#### 4.4.1. Gox@[Cu(tz)] Nanomaterials

Tumor cells metabolize glucose mainly through the glycolytic pathway for normal growth and reproduction, and cells undergoing glycolysis are less susceptible to cuproptosis than cells undergoing mitochondrial respiration; in addition, intracellular glutathione (GSH) can act as a copper chelator to inhibit cellular cuproptosis, and the level of GSH is higher in tumor cells than in normal cells [80]. Xu et al. thus hypothesized that cuproptosis in tumor cells could be promoted by depleting glucose as well as GSH in tumor cells and designed a glucose oxidase (Gox) engineered nonporous copper(I) 1,2,4-triazole ([Cu(tz)]) coordination polymer nanoplatform, namely, (GOx@[Cu(tz)]) and used it for starvation therapy-enhanced cuproptosis and photodynamic synergistic therapy. Glucose oxidase (Gox) is a natural oxidoreductase enzyme. Gox can catalyze glucose oxidation in tumor cells, consume glucose and oxygen in tumor cells, and block the energy supply of tumor cells to achieve starvation therapy. The massive depletion of GSH and glucose leads to increased sensitivity of tumor cells to Gox@[Cu(tz)]-induced cuproptosis. On the other hand, the hydrogen peroxide produced by starvation treatment can become a raw material for the occurrence of photodynamic reaction, which enhances the effect of type I photodynamic therapy and finally achieves efficient cuproptosis-based combination therapy [81]. The experimental results showed that the nano-drug could significantly inhibit the tumor growth of human bladder cancer cells (5637) in tumor-bearing mice with a tumor inhibition rate of 92.4%. The Gox@[Cu(tz)] nano drug used a combination of cuproptosis, photodynamic, and starvation therapy with high selectivity and low systemic toxicity to tumor cells, achieving an efficient combination therapy based on cuproptosis.

#### 4.4.2. Au@MSN-Cu/PEG/DSF Nano-Platforms

Cuproptosis is a novel form of programmed cell death with excellent potential for cancer therapy application. However, two pressing challenges are reducing the undesirable release of copper ions in normal tissues and maximizing the copper’s therapeutic effect at cancer sites. Zhou et al. constructed a photothermally triggered nano-platform (Au@MSN-Cu/PEG/DSF) to achieve on-demand delivery for synergistic cancer therapy [82]. It was shown that the released DSF was able to chelate in situ with Cu^2+^ to generate highly cytotoxic bis(diethyl dithiocarbamate)-Cu (CuET), which in turn caused apoptosis and formed Cu^+^ species that promoted the aggregation of toxic mitochondrial proteins, leading to cuproptosis in cells. In vivo pharmacodynamic studies demonstrated that Au@MSN-Cu/PEG/DSF synergized with photothermal therapy to kill tumor cells and inhibit tumor growth (80.1% inhibition rate). This study further advances the development of cuproptosis-based cancer treatment strategies and can provide important lessons for constructing advanced nanotherapeutic platforms. 

### 4.5. Paraptosis in Cancer

#### 4.5.1. Copper(I) Phosphide Complexes

Currently, the main treatment regimen for various solid malignancies is a combination regimen based on cisplatin (CisPt) [83]. It is well known that platinum-based anticancer drugs have serious side effects and are subject to resistance, especially for some common cancers (e.g., colon adenocarcinoma and lung adenocarcinoma [84]). Thus, researchers have been developing other potential anti-cancer metal-based compound drugs with low toxicity and high efficiency. In recent years, the copper(I) phosphide complex [Cu(thp)4][PF6] (CP) has received special attention because of its potent antiproliferative effects. CP is a monocationic copper(I) complex that is stable and highly soluble in aqueous solution, has a potent cancer cell killing ability that is more than 40 times that of CisPt, and is also able to overcome multidrug resistance. Some investigators have suggested that the cytotoxicity of CP may be related to the ability of paraptosis. Colorectal cancer (CRC) is the third most common cancer and the fourth most common cause of cancer-related death [85]. Gandin et al. investigated the inhibitory mechanism of CP in human colon cancer cell lines. They found that CP exerted a strong inhibitory effect on cells, several times higher than CisPt, without damaging normal colon fibroblasts. CP induces tumor cell death through paraptosis, and tumor cells exhibit swelling of the endoplasmic reticulum and massive vacuole formation in the cytoplasm. In conclusion, CP can potentially be a new therapeutic tool to selectively kill colon cancer cells by paraptosis action [45].

#### 4.5.2. Cu(DDC)_2_ Nanoparticles (NPs)

DSF/copper inhibits the degradation pathway of the proteasome by targeting nuclear localization protein 4 (NPL4), and copper diethyl dithiocarbamate (Cu(DDC)_2_) complexes are important components of DSF/copper to produce anticancer activity [86]. DSF also has a potential role in paraptosis, and the efficient and precise release of Cu(DDC)_2_ to tumor cells is the basis of DSF/copper anticancer. Chen et al. prepared an ideal Cu(DDC)_2_ NPs nanoparticle using SMILE technology, and this novel nanoparticle has the advantages of controlled physicochemical properties, high drug loading capacity, and stability. Experiments on the cytotoxicity and antitumor activity of MCF-7 showed that Cu(DDC)_2_ could effectively induce tumor cell death through the paraptosis pathway. Cu(DDC)_2_ binds to NPL4, blocking the corresponding pathway, inhibiting the degradation of poly-Ub protein, and thus accumulating in the endoplasmic reticulum, causing endoplasmic reticulum stress and unfolded protein response, impairing the role of mitochondria, and finally leading to paraptosis in tumor cells. Paraptosis is a promising mode of cell death, and its advanced study will promote more novel antitumor cells. It is a promising mode of cell death, and its advanced study will promote the development of more novel antitumor modalities [87].

### 4.6. Other Therapeutic Pathways

Copper is an essential trace metal element for all living organisms and is critical to vital life activities. Recent studies have revealed that tumorigenesis, neovascularization, tumor development, and metastasis are often closely related to elevated copper levels in the body. The massive discovery of copper-based antitumor complexes and nanomaterials has led to using copper-based materials as a therapeutic function in tumor therapy. Copper-based nanomaterials have become a significant hit in PTT material research due to their localized surface plasmon resonance effect and strong light absorption in the NIR region [88]. Among them, copper sulfide, a novel photothermal agent for cancer cell photothermal therapy (PTT), has received increasing attention in recent years due to the advantages of good photostability, simple synthesis, low toxicity, and low cost [89]. However, the unsatisfactory photothermal conversion efficiency of copper sulfides has limited their biological application as PTT agents. Li et al. prepared Cu_7.2_S_4_ nanocrystals (NCs) with an average size of ∼20 nm as a novel photothermal agent by a simple thermal decomposition route [90]. The amphiphilic polymer-coated Cu_7.2_S_4_-NCs exhibited good photostability and remarkable photothermal conversion efficiency up to 56.7% due to near-infrared solid absorption. Cancer cells in vitro and in vivo could effectively kill by the photothermal effect. Therefore, Cu_7.2_S_4_ NCs have significant advantages as novel PTT reagents for cancer cells. Huang et al. successfully prepared small-sized hydrophobic copper sulfide nanoparticles (CuS NPs, ≥3.8 nm in diameter) by the reaction of copper chloride with sodium diethyl dithiocarbamate (SDEDTC) in a heated oleylamine solution [91]. These CuS NPs showed strong absorption in the near-infrared (NIR) region at 700–1100 nm. After the surface coating of CuS NPs with DSPE-PEG2000, the synthesized CuS@DSPE-PEG NPs exhibited good water solubility, remarkable stability and biocompatibility, and excellent photothermal conversion upon exposure to 808 nm laser light. After intravenous administration to mice, CuS@DSPE-PEG NPs were found to passively target tumor sites and effectively ablate tumor tissue upon laser irradiation. Furthermore, CuS@DSPE-PEG NPs did not show significant toxicity in histological and blood chemistry analyses and could be efficiently excreted by metabolism. The results suggest that CuS@DSPE-PEG NPs can be used as ideal photothermal agents for cancer photothermal therapy. In conclusion, copper-based materials have great potential for future PTT applications in treating tumors.

In addition to photothermal therapy, CDT has been a Fenton chemistry-mediated tumor therapy in recent years. Still, its antitumor effect is affected by insufficient endogenous H_2_O_2_ content and the inefficient decomposition of metal oxides into catalytic ions. Despite significant progress in increasing the content of H_2_O_2_ in tumor tissues, the antitumor activity of CDT remains limited due to the inability of CDT to release enough catalytic ions to convert endogenous H_2_O_2_ into reactive oxygen species (e.g., the highly toxic hydroxyl radical ·OH) [92]. Deng et al. prepared a series of nanoparticles with adjustable acid dissociation constants (PKA) in the range of 5.2 to 6.2, loaded with H_2_O_2_ self-supplied copper peroxide, which was used to trap copper peroxide in acidic lysosomes, generating a large number of catalytic Cu ions to convert self-supplied H_2_O_2_ to ·OH via the Fenton reaction [92]. The highly reactive ·OH effectively permeates the lysosomal membrane via lipid peroxidation to kill tumor cells in a lysosomal-mediated manner. Most importantly, the Fenton reaction occurs inside the lysosome, avoiding the scavenging of ·OH by cytoplasmic antioxidants such as glutathione (GSH). Overall, this work provides a novel strategy to improve the efficacy of CDT.

The combination of CDT with copper-based materials holds great promise for tumor therapy. Liu et al. reported biodegradable amorphous copper iron tellurite nanoparticles (A-CFT NPs) encapsulated in inositol hexaphosphate (IP6) and bovine serum albumin (BSA) for efficient cancer therapy [93]. The most significant advantage of this formulation is the GSH-responsive degradation and amorphous structure, which allow the tumor-specific release of large amounts of Cu ions and the efficient utilization of Cu ions to generate -OH via Fenton-like reactions. In addition, the released Cu ions consume intracellular GSH, thus protecting OH from clearance and significantly improving CDT efficiency, which has great potential in tumor therapy. Zhou et al. prepared a copper-based multifunctional CDT agent (BiOCu_2-x_Te NSS) using polyethylene glycol-modified glutathione (GSH)-degradable Cu_2-x_Te nanosheets [94]. The obtained BiOCu_2-x_Te NSS exhibited excellent near-infrared II (NIR-II) plasma absorption and photothermal properties. BioCu_2-x_Te NSS was endocytosed by cancer cells and degraded to Cu and elemental Te due to the overexpression of GSH in cancer cells. The generated Cu and Te co-catalyzed the decomposition of endogenous H_2_O_2_ to generate ·OH, which effectively depleted GSH and disrupted the redox homeostasis of cancer cells. The weak photothermal effect of NIR-II radiation was demonstrated to promote intracellular ·OH production and the tumor accumulation of BiOCu_2-x_Te NSS. Both in vitro and in vivo studies confirmed the significant therapeutic effect of the intravenous administration of BioCu_2-x_Te NSS by the mild photothermal enhancement of CDT in the MCF-7 tumor mouse model under NIR-II irradiation. BioCu_2-x_Te NSS has the potential as an effective CDT drug with GSH-triggered degradability and good therapeutic function in tumor therapy.

Copper complexes as cancer stem cell-targeting agents are currently a hot research area. Cancer stem cells (CSCs) are a specific type of cancer cells involved in cancer development, drug resistance, and metastasis within a large tumor, with the ability of “Self-Renewal” and “Multi-Cell Differentiation” (differentiation), which is the “culprit” of cancer recurrence and metastasis [95]. DSF, an intracellular aldehyde hydrogenase (ALDH) inhibitor for the treatment of alcoholism, has been reported to target CSCs in various cancers when combined with copper. Differentiated thyroid cancers (DTCs) are common endocrine malignancies worldwide, including papillary and follicular types. Cells with high intracellular aldehyde hydrogenase (ALDH) activity are the population of CSCs in DTCs. Yung-Lun Ni et al. found that DSF/copper can target the B lymphoma Mo-MLV insertion region 1 homolog (BMI1) by inhibiting c-Myc or the E2F1-mediated expression of CSCs in DTCs [96]. Therefore, DSF/copper is a potential therapeutic agent for the future treatment of DTCs. In the presence of copper (Cu), DSF inhibits properties associated with cancer stem cells (CSCs) in breast cancer. Yoon-Jae Kim et al. investigated the mechanism of action of the DSF/Cu complex. They found that DSF/Cu complex treatment would target CSC-like cell populations, as evidenced by the inhibition of ALDH1 activity, the suppression of CD44+/CD24 and CD49f+/CD24+ subpopulations, and the subsequent impairment of mammosphere formation [97]. In the MDA-MB-231 murine xenograft model, DSF/Cu significantly reduced ALDH1A1, CD44, and phospho-STAT3 levels. Thus, DSF/Cu could serve as a cancer stem cell targeting agent that inhibits the stem cell properties of TNBC by targeting the STAT3 signaling pathway.

In summary, the diverse mechanisms of tumor cell death induction in which copper is involved will result in a richer set of preparations and therapeutic modalities for cancer patients, and the research value is very high. An overview of current copper complexes and their anticancer therapeutic applications is shown in Table 1.

## 5. Conclusions

Copper is an indispensable element in the human body and is vital in regulating essential cell cycle checkpoints. It can damage DNA and arrest the cell cycle, thereby causing apoptosis in tumor cells and significantly reducing the number of tumor cells. Copper depletion can turn off the “angiogenic switch”, which inhibits tumor neovascularization, cuts off its nutrient supply, and ultimately leads to cell death. Copper can also activate stress pathways, and this article focuses on two stress pathways, oxidative stress and endoplasmic reticulum stress. Oxidative stress refers to the occurrence of copper involved in the Haber–Weiss reaction. In contrast, endoplasmic reticulum stress disrupts intracellular protein homeostasis and changes regulatory signals into apoptosis, thereby eliminating tumor cells. Copper participates in various mechanisms of inducing tumor cell death. Cuproptosis is a newly discovered copper- and mitochondrial respiration-dependent regulatory cell death mode in which copper ions bind directly to lipid-acylated components of the tricarboxylic acid cycle pathway, leading to the abnormal aggregation of lipid-acylated proteins and the loss of iron–sulfur cluster proteins, resulting in a proteotoxic stress response that induces cellular cuproptosis. Copper involvement in the paraptosis process causes endoplasmic reticulum stress, unfolded protein responses, and massive vacuolization in the cytoplasm leading to paraptosis in tumor cells. According to different mechanisms of action, scientists have constructed different kinds of multifunctional copper complex preparations, such as new copper complexes containing naphthyl groups, hybrid copper(II) complexes, RPTDH nanoparticles, disulfiram/copper complex, Gox@[Cu(tz)] nano-drugs, copper(I) phosphide complexes, etc. These copper complex preparations effectively induce tumor cell death and show sound anti-cancer effects. Therefore, the research value of copper and copper compounds in cancer treatment is very high, and it has a vast application prospect in the future.

## 6. Outlook

As an essential trace metal for the body, copper plays a vital role in cellular metabolism. The content of copper ions in the body maintains a dynamic balance, and excess or deficiency can lead to various diseases. The critical role of copper in the human body is worthy of recognition. It can induce regulatory cell death in multiple ways. An overload or lack of copper content can lead to impaired cell function and eventually cell death, which promotes copper-targeted anti-tumor therapy research. Despite discovering the anticancer opportunity of copper and copper compounds, there are also many problems. For example, although there are many mechanisms of copper-induced cell death, this paper lists several known ones. Many means of copper-induced cell death are still unclear, in the exploratory stage, and need further research. An in-depth discussion of the role and mechanism of copper in cancer, and the improvement of more means of copper-induced tumor cell death, can provide new therapeutic targets for cancer patients. 

Although the existing studies have proved the therapeutic effect of the new copper complexes, their biocompatibility and safety must be addressed. Traditional chemotherapy is a double-edged sword. It kills normal and tumor cells, so accurately delivering copper to tumor cells without affecting other normal cells is crucial. Targeted therapy only acts on tumor cells and can avoid damage to surrounding normal tissue cells. This is a more humanized and clear advantage over traditional chemotherapy preparations. Therefore, the design and synthesis of multifunctional, targeted, and intelligent copper nano-formulations can significantly improve the availability of copper ions, change the distribution of tissues and organs, improve biosafety, and achieve efficient cancer treatment. In addition, cancer is difficult to detect and easy to metastasize, and the problem is often late when it is discovered. Therefore, more is needed to design and efficiently deliver copper preparations. Performing more research on the discovery and early detection can improve the treatment success rate. The combination of copper and diagnostic nanoparticles has dual diagnosis and treatment functions. It can realize the controllable release of drugs at the tumor site, monitor the release of active drugs, and provide timely feedback on the treatment effect. The treatment situation and the development of the disease provide an essential basis for formulating a reasonable drug regimen, which will open up a new direction for cancer copper diagnosis and treatment. The discovery of cuproptosis has elevated cancer treatment to a new dimension. The development of novel cuproptosis-induced drugs will bring new possibilities for cancer treatment, breaking the drug resistance and toxic side effects accompanying therapy with traditional cell death modalities and achieving higher anti-tumor outcomes. In conclusion, the in-depth research and exploration of the anti-cancer mechanism of copper can not only enrich tumor treatment methods but also provide more theoretical guidance and solutions for clinically refractory malignant tumors in combination with traditional treatment.

## Figures and Tables

**Table 1:** Summary of current copper complexes and their anticancer therapeutic applications.

Copper Complexes	Mechanisms	Evaluation Model	Function Description	Ref.
RGD-PEG-b-PGA-g-(TETA-DTC-PHis) (RPTDH)	Antiangiogenesis	4T1, MCF-7 MDA-MB-231, and human umbilical vein endothelial cells	Chelated copper ions inhibit the angiogenic ability of breast cancer cells and suppress their growth and metastasis	[73]
VEGF73-101/Cu(II)complex	Antiangiogenesis	Human umbilical vein endothelial cells (HUVEC)	Inhibit angiogenesis and promotes apoptosis in the cell membrane model	[77]
Disulfiram/Copper Complex	Activate signal pathway	HL60/ADM cells	Activation of the MAPK pathway induces apoptosis	[55]
	ROS generation and cytotoxicity	MKN-45 and BGC-823 cells	Provide an oxidative environment and induce programmed cell death	[59]
	Induced DNA damage	MKN-45 and BGC-823 cells	Inducing DSBs leads to cancer cell death	[60]
	Regulating energy metabolism	MKN-45 and BGC-823 cells	Inhibit the glycolytic pathway of gastric cancer cells and also inhibit the mitochondrial respiratory pathway of gastric cancer cells	[61,62]
Cu1 complex	endoplasmic reticulum stress	A549 cancer cells	Increased permeability of the ER membrane leads to ER-mediated apoptosis	[33]
[Cu(L^1^)(phen)](ClO_4_)	The arrest of cell cycle	Human breast cancer cell line MDA-MB-231, rat myoblast L6	Block the growth of S-phase cells and inhibit DNA synthesis	[68]
[Cu(L^2^)(bpy)](ClO_4_)	The arrest of cell cycle	Human breast cancer cell line MDA-MB-231, rat myoblast L6	MDA-MB-231 cells showed higher activity, arrested cell growth and arrested it in the S phase, and inhibited cell DNA synthesis	[68]
[(H_2_L_2_)Cu(μ-Cl)2Cu(H_2_L_2_)]Cl_2_·5H_2_O [(H_2_L_4_)CU(μ-CL)2CU(H_2_L_4_)]CL_2_·6H_2_O	The arrest of cell cycle	Human leukemia cell lines THP-1 and U937	Arrest the growth of G1 phase cells, leading to cell apoptosis	[64]
[(HL_1_)Cu(μ-Cl)2Cu(HL_1_)]Cl_2_·H_2_O [(HL_3_)Cu(μ-Cl)2Cu(HL_3_)]Cl_2_·4H_2_O	The arrest of cell cycle	Human leukemia cell lines THP-1 and U937, PBMC cells	It showed higher activity against THP-1 and U937, arrested cell growth in the G1 phase, and led to cell apoptosis	[64]
Tripyridine copper (II) complex	The arrest of cell cycle	Hepatocellular carcinoma BEL-7402	By inhibiting the activity of cyclin E-cdk2 and increasing the expression levels of p53 and p21, BEL-7402 cells were arrested in the G1 phase, thus inhibiting cell proliferation	[69]
[Cu_2_L^1–5^(ClO_4_)](ClO_4_)	The arrest of cell cycle	A431 cell	Block DNA replication and inhibit tumor cell growth	[65]
Pyridoxal Amino-Thiourea/Copper complex	Promotes cell cycle arrest and inhibits cell division	SK-BR-3 cell	Inhibits cell division by selectively inhibiting the activity of topo-I and topo-II alpha	[79]
Gox@[Cu(tz)]	Cuproptosis	MCF-7 and 5637 cells	Treatment by a combination of cuproptosis, photodynamic and starvation therapy	[81]
Au@MSN-Cu/PEG/DSF	Cuproptosis	4T1 cells	Synergistic killing of tumor cells through cuproptosis and photothermal therapy	[82]
CP	Paraptosis	HFF-1, MRC-5 and CCD-18Co cells	Paraptosis induce tumor cell death	[45]
Cu(DDC)_2_NPs	Paraptosis	MCF-7 cells	induces paraptosis in tumor cells by causing endoplasmic reticulum stress and unfolded protein responses	[87]
Cu_7.2_S_4_ nanocrystals	PTT	Cancer cells in mice	Good photostability and remarkable photothermal conversion efficiency up to 56.7%. Cancer cells in vitro and in vivo can be effectively killed by the photothermal effect	[90]
CuS@DSPE-PEG NPs	PTT	Cancer cells in mice	Passive targeting of tumor sites and effective ablation of tumor tissue under laser irradiation	[91]
A-CFT NPs	CDT and Fenton reaction	4T1 and L-O2 cells	Release large amounts of Cu ions and use them efficiently to produce ·OH by a Fenton-like reaction	[93]
BiOCu_2-x_Te NSS	PTT and Fenton reaction	MCF-7 cell	Catalytic decomposition of endogenous H_2_O_2_ to generate ·OH disrupts the redox homeostasis of cancer cells	[94]
DSF/Copper complex	Inhibition of thyroid globule formation and CSC activity	K1 and WRO cells	Inhibition of B-lymphoma Mo-MLV insert 1 homolog expression and cancer stem cell activity to induce its antidifferentiated thyroid cancer effect	[96]
DSF/Copper complex	—	MDA-MB-231 and 4T1 cells	Inhibition of aldehyde dehydrogenase activity, induction of apoptosis in breast cancer stem cells	[97]

## Data Availability

Data sharing not applicable.
